# SYNBIOCHEM–a SynBio foundry for the biosynthesis and sustainable production of fine and speciality chemicals

**DOI:** 10.1042/BST20160009

**Published:** 2016-06-09

**Authors:** Pablo Carbonell, Andrew Currin, Mark Dunstan, Donal Fellows, Adrian Jervis, Nicholas J.W. Rattray, Christopher J. Robinson, Neil Swainston, Maria Vinaixa, Alan Williams, Cunyu Yan, Perdita Barran, Rainer Breitling, George Guo-Qiang Chen, Jean-Loup Faulon, Carole Goble, Royston Goodacre, Douglas B. Kell, Rosalind Le Feuvre, Jason Micklefield, Nigel S. Scrutton, Philip Shapira, Eriko Takano, Nicholas J. Turner

**Affiliations:** * BBSRC/EPSRC Synthetic Biology Research Centre for Fine and Speciality Chemicals (SYNBIOCHEM), Manchester Institute of Biotechnology, The University of Manchester, Manchester, U.K.; † SYNBIOCHEM Platform/Theme Lead, School of Chemistry, The University of Manchester, Manchester, U.K.; ‡ SYNBIOCHEM Data Lead, School of Computer Science, The University of Manchester, Manchester, U.K.; § SYNBIOCHEM, Manchester Institute of Biotechnology, The University of Manchester, Manchester, U.K.; ║ SYNBIOCHEM, The University of Manchester, Manchester, U.K.; ¶ SYNBIOCHEM Responsible Research Innovation Lead, Alliance Manchester Business School, The University of Manchester, Manchester, U.K.

**Keywords:** biocatalysis, fine chemicals, metabolic engineering, synthetic biology, systems biology

## Abstract

The Manchester Synthetic Biology Research Centre (SYNBIOCHEM) is a foundry for the biosynthesis and sustainable production of fine and speciality chemicals. The Centre's integrated technology platforms provide a unique capability to facilitate predictable engineering of microbial bio-factories for chemicals production. An overview of these capabilities is described.

The Manchester University Synthetic Biology Research Centre (SBRC), SYNBIOCHEM (http://synbiochem.co.uk), is focused on delivering Synthetic Biology (SynBio) solutions that provide innovative, fast, predictable and robust production routes for diverse fine and speciality chemicals underpinned by Responsible Research and Innovation (RRI). By harnessing the power of predictive SynBio methods, SYNBIOCHEM is driving next-generation sustainable manufacturing processes that are appropriate and commercially relevant for scale-up across many industrial sectors (e.g. healthcare, energy, agrichemicals, green chemistry, pharmaceuticals, novel materials and bioremediation).

SYNBIOCHEM is located in the Manchester Institute of Biotechnology (MIB; http://www.mib.ac.uk/) and builds on the MIB's core strategy of uniting teams of interdisciplinary scientists and industrial partners to deliver challenge-led innovation in bio-based chemicals and broader industrial biotechnology sectors. SYNBIOCHEM also builds on a long-term vision and commitment by the University of Manchester in sustainable Industrial Biotechnology, leveraging and integrating world-leading capabilities in MIB Centres of Excellence including: Biocatalysis, Biotransformation and Biocatalytic Manufacture (CoEBio3); Manchester Centre for Biophysics and Catalysis (MCBC); Manchester Centre for Integrative Systems Biology (MCISB) and the Michael Barber Collaborative Centre for MS (MBCMS). SYNBIOCHEM was established in 2014 funded jointly by the U.K. Biotechnology and Biological Sciences Research Council, the U.K. Engineering and Physical Sciences Research Council and The University of Manchester. Led by three directors (Nigel Scrutton, Eriko Takano and Nick Turner) the Centre unites a complementary team of SynBio researchers at Manchester in pursuit of SynBio solutions that will deliver wider access to chemical diversity and more rapid and predictable delivery of chemical targets for scalable production/manufacturing. The interdisciplinary team includes not only chemists, biologists and computer scientists, but also investigators from the Manchester Institute of Innovation Research and the School of Social Sciences contributing insight of RRI relevant to the Centre activities. A key challenge includes consideration of the societal impact of SYNBIOCHEM research activities to ensure that the Centre anticipates, prepares for and, if necessary, mitigates the impact of SynBio technology in the wider society, economy and environment [1].

SYNBIOCHEM has established state-of-the-art and fully integrated Design/Build/Test technology platforms for bio-based chemicals production (Figure 1). The platforms include innovative DESIGN platforms for the computational modelling and design of biological parts, devices and chassis; state-of-the-art high-throughput and automated BUILD capabilities for the rapid assembly of engineered microbial systems; and a suite of advanced analytical TEST technologies, including mass spectrometers for sensitive targeted chemical detection and untargeted metabolomics, and picodroplet/fermentation platforms for production and analysis from the single cell through to the 1 l scale. The technology platforms are supported by a comprehensive fully shared standards-compliant data management platform that ranges from laboratory information management system (LIMS) to notebooks to cross project cataloguing that will be openly available. The SYNBIOCHEM platforms are supported by an expert team of Senior Experimental Officers to implement the Centre's science programmes, which are currently focused on the engineering of microbial bio-factories for the sustainable production, scale-up and diversification of terpenoids, flavonoids and alkaloids.

**Figure 1:**
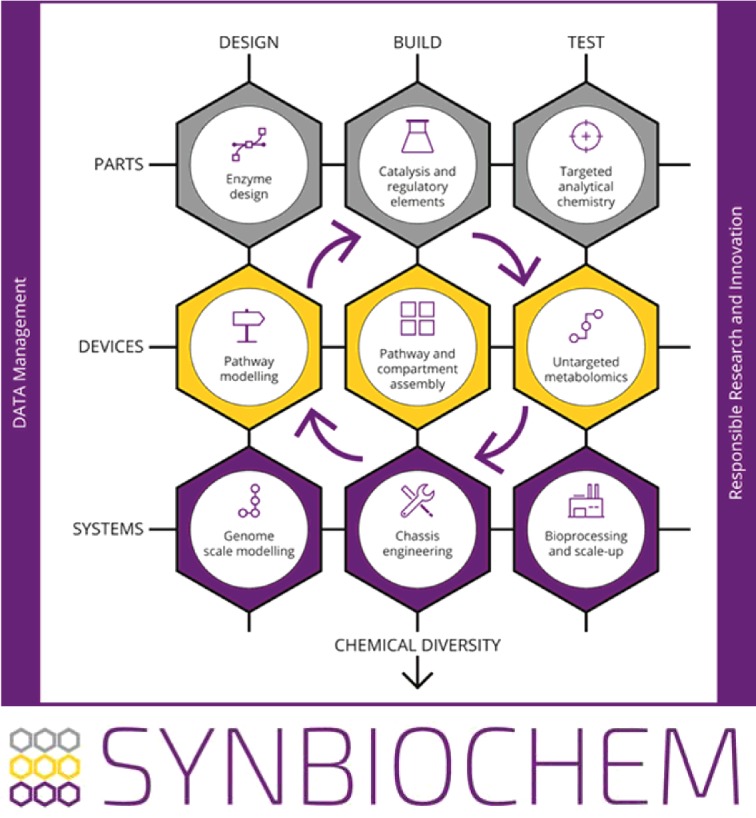
Schematic of the SYNBIOCHEM synthetic biology foundry Schematic of the foundry concept established in SYNBIOCHEM that enables iterative Design, Build and Test workflows for the engineering of new biological parts (e.g. enzymes and regulatory components), devices (e.g. pathways and cellular compartments) and systems (microbial chassis and processes).

SYNBIOCHEM has an open and collaborative culture with a strong emphasis on building academic and industry partnerships, both at the national and international levels. Industrial foresight and awareness coupled to translation of SYNBIOCHEM discovery science and technology towards commercial exploitation are at the core of the Centre's strategy. The Centre hosts regular open meetings in a variety of formats, enabling one-to-one discussions with industrial partners, the development of new collaborative projects with external stakeholders, and through an industry club for joint supervision of research studentships in projects that address scientific problems of interest to the club members. Early SYNBIOCHEM programmes have resulted in protection of new intellectual property for the development of tools and microbial factories/hosts relevant to the production of pravastatin [2], biosynthetic menthol [3] and propane [4], the development of new regulatory components (e.g. riboswitches [5]) and the discovery of new biocatalysts for chemicals production that provide new routes to alkenes [6,7] and enantiopure amines [8]. This is supported by unique technology innovations in DNA design and assembly [9,10] that underpin the rapid construction and evolution of new parts and pathways through, for example new approaches to directed evolution [11]. Many of these discoveries are subject to new SYNBIOCHEM patent submissions and, in one case, have already supported the formation of an early spinout company from the Centre.

In summary, SYNBIOCHEM is addressing major challenges in SynBio through a foundry concept and an open collaborative ethos with external partners. The Centre's integrated technology platforms provide a unique capability to facilitate predictable engineering of microbial bio-factories for chemicals production. Further information about the Centre and routes to collaboration can be obtained from the SYNBIOCHEM website: http://synbiochem.co.uk.

## Abbreviations

MIB: Manchester Institute of Biotechnology
RRI: Responsible Research and Innovation
SynBio: Synthetic Biology
